# Transcriptome and Genome Size Analysis of the Venus Flytrap

**DOI:** 10.1371/journal.pone.0123887

**Published:** 2015-04-17

**Authors:** Michael Krogh Jensen, Josef Korbinian Vogt, Simon Bressendorff, Andaine Seguin-Orlando, Morten Petersen, Thomas Sicheritz-Pontén, John Mundy

**Affiliations:** 1 Department of Biology, University of Copenhagen, Copenhagen, Denmark; 2 Center for Biological Sequence Analysis, Department of Systems Biology, Technical University of Denmark, Lyngby, Denmark; 3 National High-throughput DNA Sequencing Centre, Copenhagen, Denmark; University of Padova, ITALY

## Abstract

The insectivorous Venus flytrap (*Dionaea muscipula*) is renowned from Darwin’s studies of plant carnivory and the origins of species. To provide tools to analyze the evolution and functional genomics of *D*. *muscipula*, we sequenced a normalized cDNA library synthesized from mRNA isolated from *D*. *muscipula* flowers and traps. Using the Oases transcriptome assembler 79,165,657 quality trimmed reads were assembled into 80,806 cDNA contigs, with an average length of 679 bp and an N50 length of 1,051 bp. A total of 17,047 unique proteins were identified, and assigned to Gene Ontology (GO) and classified into functional categories. A total of 15,547 full-length cDNA sequences were identified, from which open reading frames were detected in 10,941. Comparative GO analyses revealed that *D*. *muscipula* is highly represented in molecular functions related to catalytic, antioxidant, and electron carrier activities. Also, using a single copy sequence PCR-based method, we estimated that the genome size of *D*. *muscipula* is approx. 3 Gb. Our genome size estimate and transcriptome analyses will contribute to future research on this fascinating, monotypic species and its heterotrophic adaptations.

## Introduction

Darwin was fascinated by the unusual adaptations of carnivorous plants during his often frustrating studies of the evolution of flowering plants, which he referred to as an ‘abominable mystery’ [1,2]. Darwin’s treatise on insectivorous plants noted that the Venus flytrap (*Dionaea muscipula*) was ‘one of the most wonderful of the world’ [3]. Studies of carnivorous plants have continued since Darwin’s time. Attention has focused on the biogeography and phylogenetics of the only two carnivorous species with snap traps, *D*. *muscipula* and the aquatic waterwheel *Aldrovanda vesiculosa* [4–6]. The habitat of *D*. *muscipula* is damp pine savannas of southeastern North America, and it is considered a relic species with a narrow, endangered distribution of less than 300 km^2^ [4]. *A*. *vesiculosa* is also considered a relict, earlier widely distributed in Europe, Africa, India, Japan, and Australia, yet now confined to fewer than 36 localities mostly in Europe and Russia [7].

Earlier phylogenetic studies demonstrated that carnivory occurs in several flowering plant lineages [8,9], and it was thought that the snap traps of *A*. *vesiculosa* and *D*. *muscipula* evolved independently. However, Cameron *et al*. [4] showed that *A*. *vesiculosa* and *D*. *muscipula* evolved as monotypic sister genera from a sundew-like ancestor. While the habitat of *A*. *vesiculosa* is similar to that of many aquatic carnivorous bladderworts (*Utricularia* spp.), the snap traps of *D*. *muscipula* and *A*. *vesiculosa* are unique in having a single evolutionary origin and narrow ecological distributions [1].

An understanding of the molecular adaptations to plant carnivory has also been sought via genome size estimates. Genome sizes vary more than 2,300-fold among angiosperms, from that of *Paris japonica* (2*n* = 12, 1C = ~149 Gbp) [10] to that of carnivorous *Genlisea margaretae* (2*n* = ~40, 1C = ~63 Mbp) [11]. The biological significance of this massive variation is puzzling. Carnivorous plants are found in at least five, genetically poorly described orders [12]. The lack of molecular tools and genetic information, however, has not hampered phenotypic and ecological studies of the orders with carnivorous members [1,13], and comparative genomic analyses may clarify some of their traits. Within the *Lentibulariaceae*, Greilhuber *et al*. identified ~24-fold variation in genome sizes among *Genlisea* and other family members [11]. Also, large variations in ploidy levels and chromosome sizes have been reported within the carnivorous *Droseraceae* [14]. Rogers *et al*. reported genome estimates for two carnivorous pitcher plants, *Sarracenia purpurea* and *Sarracenia psitticina*, to be larger than 3.5 Gb [15]. Thus, the genome contents of carnivorous plants seem to be extremely variable, and the larger genomes tend to have many repetitive sequences and transposable elements [15].

An important complement to genome size analyses comes from transcriptome data. Both transcriptome and genome sequence data are needed to understand the physiological and genetic basis of the snap trap and to identify genes selected during its evolution [16]. To this end, deep sequencing [17,18] is beginning to reveal certain aspects of the evolution of carnivory. To date transcriptome data for the bladderwort *Utricularia gibba* has been published [19]. Furthermore, Srivastava *et al*. have reported the deep-sequencing of two *Sarracenia* species [20], thereby providing important information on the events of genome duplication and speciation within the genus *Sarracenia*. Finally, Schulze *et al*. used transcriptome data to delineate the protein composition of the digestive fluid of *D*. *muscipula* [21]. Altogether, such studies clarify aspects of the molecular physiology associated with the carnivorous syndrome.

In the present study, we sequenced the transcriptome of *D*. *muscipula*, using a mixed-tissue sample for cost-effective, next generation sequencing of a normalized cDNA library. Transcriptome sequences were assembled into contigs and functional analyses performed. From this a large number of transcripts related to catalytic activities were identified. This high-throughput data set is the first available for a member of the largest family of carnivorous plants (Droseraceae). Our data provide a public resource for unveiling mechanistic features of the carnivorous syndrome such as attraction, trapping and digestion. Moreover, our *D*. *muscipula* genome size estimate, based on quantitative PCR of a single copy sequence, is the first for a member of the sundew family in the order Caryophyllales.

## Materials and Methods

### Plant material

For nuclear genome estimates, 1 g of freshly harvested flowers, petioles and traps were used from *D*. *muscipula* and *Arabidopsis thaliana* (Col-0). *D*. *muscipula* plantlets were purchased from Horticulture Lammehave A/S (Ringe, Denmark).

### Genomic DNA extraction

DNA was extracted from *D*. *muscipula* and *A*. *thaliana* as described for *Drosera rotundifolia* by Bekesiova *et al*. [23] with modifications for extraction from the more succulent and recalcitrant *D*. *muscipula*. After tissue grinding, cells were lysed in 6 ml CTAB-buffered N-lauryl sarcosine (5%) with 2 ul 2-mercaptoethanol and 0.3 g polyvinylpyrrolidone (PVPP), (MW = 360,000, Sigma) per ml lysis buffer, and incubated 1 hr at 65°C in a water bath. The lysate became more viscous as the solution was cooled at room temperature for 10 min before extraction with 1 x volume of 24:1 chloroform:isoamoyl alchohol (IAA). The sample was centrifuged at 13,000 RPM for 10 min at 4°C. A 5-ml pipette was used to gently transfer the upper aqueous phase to new tubes and DNA was precipitated over-night at -20°C using 0.1 volume of 3 M Na-acetate (pH 5.2) and 2.5 volume ethanol. DNA was collected by centrifugation (20 min, 13,000 RPM, 4°C), the pellet washed in 70% ethanol and centrifugation repeated. The pellet was briefly air-dried at room temperature before being gently dissolved in 1 ml TE (pH 7.5). Due to high absorbance at 230 nm, a second purification was done. 1^st^, resuspended DNA was treated for 1 hr at 37°C with 50 ug/ml RNase A (Sigma) and 50 units/ml RNase T1 (Fermentas). Proteinase K (150 μBg/ml) was then added for another hour at 37°C. Subsequently, 1 x volume of CTAB buffer was added and the solution incubated 1 hr at 65°C. 1 ml of chloroform:IAA (24:1) was then added and mixed. After centrifugation (10 min, 13,000 RPM, 4°C), the supernatant was precipitated over-night at -20°C with 0.1 volumes of 3 M Na-acetate (pH 5.2) and 2.5 volumes ethanol. DNA was collected by centrifugation as above, the pellet washed in 70% ethanol and centrifugation repeated. The pellet was air-dried for 30 min at room-temperature and resuspended in TE (pH 7.5) or water. DNA purity and concentration were measured on a nanodrop 1000 (Thermo scientific).

### mRNA Isolation

Total RNA was extracted from 1.5 g fresh weight each of *D*. *muscipula* flowers and traps using an optimized urea-based protocol. For a single extraction, 0.1 g (approx. equivalent to 1 medium-sized trap) tissue was flash-frozen in liquid nitrogen and ground with 0.03 g of PVPP. This powder was transferred to a pre-warmed (65°C) microcentrifuge tube containing 700 ul of RNA extraction buffer (2% CTAB (w/v), 2% PVP K25 (w/v), 100 mM Tris-HCl (pH 8.0), 25 mM sodium-EDTA (pH 8.0), 2.0 M NaCl, 2% (w/v) β-mercaptoethanol and vigorously shaken. The suspension was then centrifuged for 2 min at 13,000 RPM to pellet debris, and the supernatant transferred to a new tube. Subsequent steps were at 4°C. The suspension was extracted with 600 ul chloroform: IAA (24:1), and phases separated by centrifugation (10,000 RPM, 10 min.). The aqueous phase was then re-extracted with 500 μl phenol: chloroform: AA (25:24:1) and centrifuged. RNA in the aqueous phase was precipitated overnight with 0.25 volumes (125 ul to 500 ul) 10 M LiCl added with gentle mixing, then pelleted by centrifugation (10,000 RPM, 20 min). RNA was resuspended in 100 ul of DEPC-treated water, then re-precipitated with 250 μl 20% 1M sodium acetate (pH 5.2) and 80% EtOH and incubated 1 hr at -70°C. Following centrifugation, the pellet was gently washed in 70% RNase-free EtOH, centrifuged, and resuspended in 30 ul DEPC-treated water. Total RNA was RQ1 DNase treated (Promega), and mRNA isolated from 2–3 mg of trap and flower total RNA using PolyATtract (Promega) according to the manufacturer’s description.

### cDNA library construction, sequencing and assembly

The MINT kit (Evrogen) was used for 1^st^-strand cDNA synthesis with 400 ng mRNA from each sample. Following evaluative PCR, a full-sized pre-saturation synthesis of ds-cDNA was prepared for both tissues using Encyclo PCR (Evrogen). cDNA was purified using QIAquick (Qiagen) and concentration measured using Qubit (Invitrogen). Samples were then pooled in a 1:4 ratio of trap:flower cDNA to a total of 1 ug cDNA for normalization using duplex-specific nuclease [33]. Normalization was evaluated by PCR using Evrogen PCR adaptor-specific primer M1, and full-size cDNA amplification performed. A total of 4 ug cDNA was subsequently fragmented using a Bioruptor (Diagenode) and MinElute (Qiagen) purified prior to library building. The NEBNext Quick DNA library kit (New England Bioloabs) was used for library building with 0.5 ug fragmented cDNA and 1 ul of 15 uM InPE adaptor (Illumina). Following another MinElute step, we indexed (6-bases) and amplified the library 10x with Illumina standard primers (InPE1.0 and InPE2.0). Finally, the library was evaluated by gel electrophoresis and a gel piece containing 270–320 bp fragments was isolated and QIAquick purified (Qiagen).

The library was sequenced using Illumina HiSeq2000 technology with 100 bp single-end reads at the National High-throughput DNA Sequencing Centre, University of Copenhagen. All sequenced reads are uploaded to National Center of Biotechnology Information Sequence Read Archive (NCBI SRA) and can be accessed with the accession number SRX312294. Prior to *de novo* assembly using Oases [34], adaptor sequences were trimmed and low quality reads removed (Phred quality score < 20) by genobox (https://github.com/srcbs/GenoBox) including the FASTX-Toolkit (http://hannonlab.cshl.edu/fastx_toolkit/). The Oases transcriptome assembly can be found in S1 Table. To quality assess the transcriptome assembly, contigs were aligned to putative low- and high-abundant transcript genes (sequence gis in S2 Table) by BLASTn (E-value < = 1E-5), and 10 contigs of varying size were selected for confirmation. Primers were designed using Primer3 [35]. Sequences for primers and contigs are in S3 Table.

### Functional annotation

Assembled transcriptome contigs were aligned to NCBI non-redundant protein databases (nr, May 2013) using BLASTx (E-value < = 1E-05, bit score > = 50). Gene names and annotation were assigned to the corresponding contig based on the best BLASTx hit. The BLAST results for the best hits can be found in S2 Table. Transcripts for each locus were scanned with InterProScan (http://www.ebi.ac.uk/Tools/pfa/iprscan/) and integrated protein databases with default parameters. The GO terms associated to the transcriptome contigs were retrieved to describe genes in the categories of cellular components, molecular function and biological process. The functional gene annotation for *Arabidopsis* was retrieved from The Arabidopsis Information Resource, version TAIR10 [36].

### Assembly assessment and full-length cDNA identification

Assembled contigs were aligned to non-redundant protein databases with a cut-off E-value of 1E-5, and putative full-length cDNA sequences and ORFs were identified by TargetIdentifier [22]. cDNA sequences are classified as full-length if the following criteria were fulfilled (1) the sequence has a start codon with a downstream stop codon or (2) the sequence has a stop codon and an in-frame start codon is detected prior to the 10^th^ codon of the aligned subject sequence. For comparison of the *D*. *muscipula* open reading frames to other plant proteins, contigs were aligned (standard parameters, E-value < 1E-5) to 8 RefSeq and Ensembl proteins, including *Arabidopsis thaliana*, *Brachypodium distachyon*, *Oryza sativa*, *Physcomitrella patens*, *Ricinus communis*, *Vitis vinifera*, *Solanum lycopersicum* and *Zea mays*.

### qPCR estimate of genome size

Sequencing of the transcriptome of traps and flowers of *D*. *muscipula* gave 80,806 contigs. A long unique sequence with good coverage was chosen for primer design as shown in S4 Table. The sequence had 86% identity to the Arabidopsis *ACT7* gene (AT5G09810). Primers were from MWG Biotech (Ebersberg), and the qPCR-based analysis of genome size was performed according to Wilhelm *et al*. [24] using a Bio-Rad iCycler (Bio-Rad). The genome size, described as gametic nuclear DNA contents (‘C-values’), either in units of mass (picograms, where 1 pg = 10^-12^ g) or in number of base pairs (where 1 pg DNA = 0.978 × 10^9^ bp;[37]) was calculated by dividing the mass of sample DNA by the copy number determined for single copy genes.

## Results and Discussion

### Transcriptome Sequencing and Assembly of *D*. *muscipula*

To analyze the transcriptome of *D*. *muscipula*, a normalized library of mixed mRNAs from traps and flowers was sequenced using Illumina HiSeq2000 technology. A total of 81,329,943 single-end 100-bp reads were generated. After removal of ambiguous nucleotides and low-quality sequences (Phred quality score < 20), a total of 79,165,657 cleaned reads (97.3%) were obtained. These raw transcriptome sequences were deposited in the NCBI SRA database (accession SRX312294), and quality controlled reads assembled. As shown in Table 1, the assembly combined the 79,165,657 reads into 80,806 contigs, with an average length of 679 bp and an N50 length of 1,051 bp.

**Table 1:** Statistics of transcriptome sequencing and assembly of *D*. *muscipula*.

**Sequencing**	# Reads (93-bp single-end)	81,329,943
	Total bases	7.56 Gb
	# Cleaned reads	79,165,657
**Assembly**	Numbers of contigs	80,806
	Max contig length	7,545 bp
	Min contig length	100 bp
	N50 length	679 bp
	Mean contig length	1051 bp

**Fig 1:**
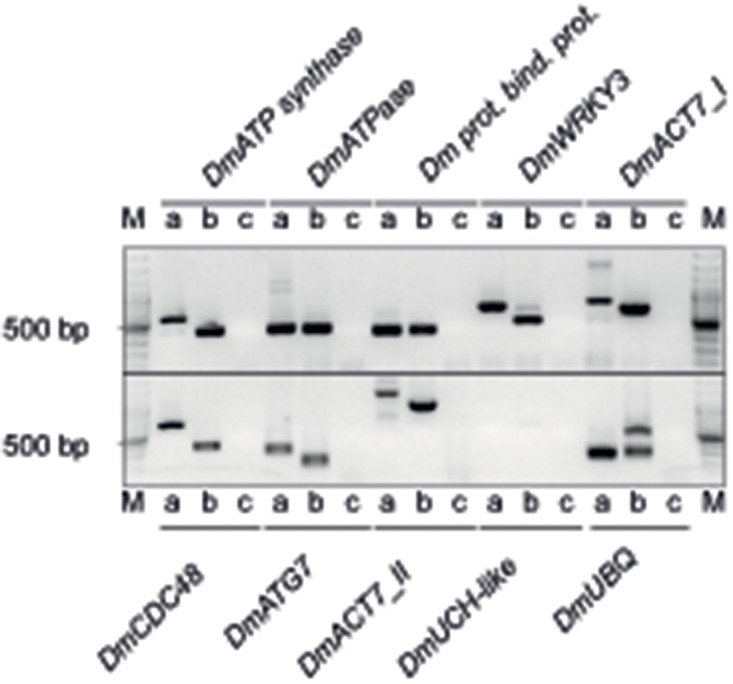
PCR assembly validation. Contigs assembled from 93 bp single-end reads were validated using standard PCR. A: genomic DNA, B: First-strand cDNA synthesis with reverse transcriptase, C: First-strand cDNA synthesis without reverse transcriptase. M: 100 bp O’GeneRuler. For primer and contig sequences, see S1 Table.

To quality assess contig assemblies and validate our normalization procedure, we selected 10 contigs for PCR-based validation. These contigs were selected based on alignment annotation to putative low- and high-abundant transcript genes. Actin and ubiquitin sequences were included as high-abundant mRNA transcripts, while transcription factor sequences were included as putative low-abundant mRNA transcripts. Also, primers for validation of assembly were designed to target a range of contig sizes. Using an independent biological replicate cDNA template of *D*. *muscipula* traps and flowers, we then validated transcript assemblies of putative low- and high- abundant transcripts ranging from 247–1,366 bp (Fig 1 and S3 Table), including both. Expected amplicon sizes were obtained from all ten contigs, although no genomic amplicon was obtained for *DmUCH-like* (S3 Table). This confirmed that assembly using Oases was reliable, and that our normalization procedure identified transcripts with varying abundances.

### Functional Annotation

Assembled contigs were aligned to the NCBI non-redundant (nr, May 2013) protein database for functional annotation by BLASTx with an E-value cut-off of 1e-5. A total of 42,656 contigs had significant hits, corresponding to 17,047 unique protein accessions in the nr protein database (Table 2).

**Table 2:** Summary of BLASTx search results of *D*. *muscipula* transcriptome.

Database	*D*. *muscipula* hits	Unique protein hits	% of total unique proteins
**nr**	42,656	17,047	
**Refseq/Ensembl**			
*Arabidopsis thaliana*	41,422 (51.3%)	13,469	38.1% (13,469/35,378)
*Brachypodium distachyon*	39,962 (49.4%)	**11,795**	48.8% (11,795/24,689)
*Oryza sativa*	39,353 (48.7%)	**11,506**	40.1% (11,506/28,705)
*Physcomitrella patens*	34,084 (42.2%)	**9,390**	26.1% (9,390/35,936)
*Ricinus communis*	41,839 (51.7%)	**12,279**	39.1% (12,279/31,344)
*Vitis vinifera*	43,634 (53.9%)	**12,837**	53.8% (12,837/23,877)
*Zea mays*	35,229 (43.6%)	**10,194**	45.1% (10,194/22,588)
*Solanum lycopersicum*	42,489 (52.6%)	**13,152**	59.8% (13,152/26,408)

From a total of 80,816 contigs, 42,656 have a RefSeq hit, corresponding to 17,047 unique protein entries. Total number and unique hits from a BLASTx against RefSeq entries for 8 other plant species is also presented. The percent of total unique proteins is based on the current number of RefSeq entries for the individual species.

**Fig 2:**
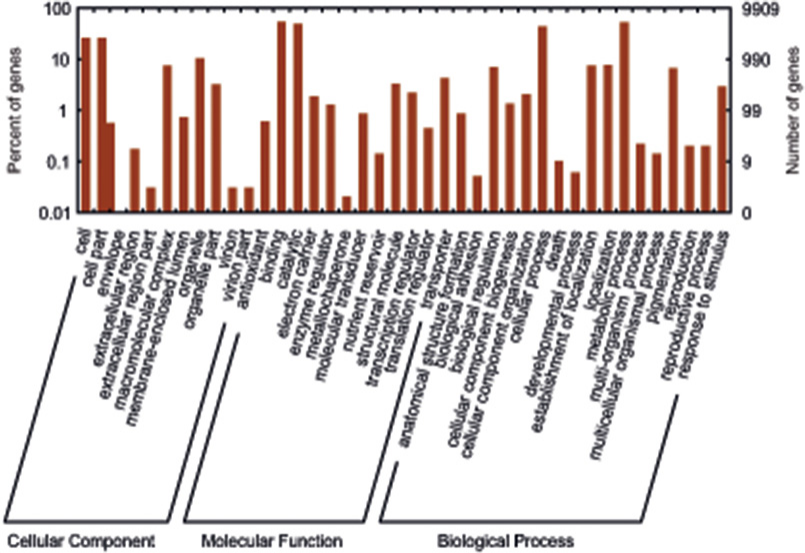
Gene Ontology (GO) categories of the unigenes. Distribution of the GO categories assigned to the *D*. *muscipula* transcriptome. Unique transcripts (unigenes) were annotated in three categories: cellular components, molecular functions, and biological processes.

Functional analysis was conducted on these 17,047 unique proteins using InterProScan (http://www.ebi.ac.uk/Tools/pfa/iprscan/) and integrated protein databases with default parameters. A total of 9,909 unique proteins were assigned to at least one gene ontology (GO) term for describing biological processes, molecular functions and cellular components. GO annotations were plotted with WEGO (http://wego.genomics.org.cn) (Fig 2). Briefly, in the cellular component division, genes related to cell parts and macromolecular complexes (2,588 (26.3%) GO:0044464 and 746 (7.6%), GO: 0032991, respectively) are highly represented. Interestingly, in contrast to other plants, *D*. *muscipula* also has genes related to a virion part (3 (0.1%), GO:0044423). For the molecular function division, a large abundance of genes are related to binding and catalytic activity (5348 (54.4%) GO:0005488 and 4847 (49.3%) GO:0003824, respectively). Also, antioxidant (56 (0.6%) GO:0016209) and electron carrier activities (184 (1.9%) GO:0009055) are represented. For the biological process division, genes involved in cellular (4,285 (43.6%), GO:0009987) and metabolic processes (5,136 (52.2%), GO: 0008152) are highly represented, including the child term of establishment of localization (733 (7.4%), GO:0051234). In contrast, genes associated with developmental and multicellular organismal processes were lowly represented (6 (0.1%), GO:0032502; and 14 (0.1%) GO:0032501, respectively) compared to full-genome annotations for *Arabidopsis* (15% and 15.5%, respectively). This may well reflect the limited tissues and developmental stages sampled here for *D*. *muscipula*. The complete GO annotation results are in S5 Table.

### Assessment of Transcriptome Assembly

Assembled transcript contigs were aligned to all RefSeq entries (May 2013) for a moss (*Physcomitrella patens*), the angiosperms grape (*Vitis vinifera*), *Arabidopsis thaliana*, tomato (*Solanum lycopersicum*), *Brachypodium distachyon*, rice (*Oryza sativa*), maize (*Zea mays*), and the monotypic oil plant *Ricinus communis* using BLASTx with an E-value cutoff of 1e-5 (Table 2). Cross-species sequence similarity identified most hits in grapes, tomatoes, oil plants and *Arabidopsis*. Considering unique protein hits, the *D*. *muscipula* transcriptome from our normalized mixed-tissue cDNA library targeted almost 60% of the tomato and more than 50% of the grape Refseq data. Likewise, almost 50% of the *Brachypodium* RefSeq data was uniquely aligned to individual *D*. *muscipula* contigs. For *Arabidopsis*, 13,469 unique protein hits were identified, covering more than a third of the *Arabidopsis* Refseq protein entries. These numbers represent underestimates of the minimal number of *D*. *muscipula* genes expressed in flowers and traps. Apart from tissue-specificity, it is possible that many *D*. *muscipula* unique protein hits could not be aligned to RefSeq hits because they represent untranslated regions (UTRs) and/or non-coding RNAs (ncRNAs). Better characterization of the *D*. *muscipula* transcriptome would require a more complete set of transcriptome data from various tissues across a longer developmental span.

### Full-Length cDNA prediction

**Fig 3:**
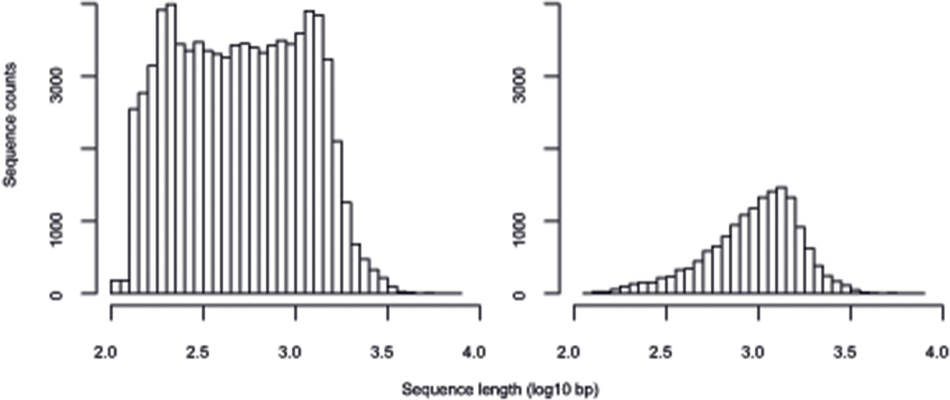
Contig size distribution. Transcriptome assembly contig size distribution of all contigs (left) and predicted full-length contigs (right).

Full-length cDNAs are important resources for many applications, including reverse genetic and evolutionary studies. To search for potentially full-length cDNAs with complete open-reading frames (ORFs) in the assembled *D*. *muscipula* transcriptome, all contigs were analyzed by TargetIdentifier [22]. A total of 15,547 full-length sequences were identified from the assembly. The size distribution of full-length sequences compared to that of the total 80,806 cDNA contigs is shown in Fig 3. In contrast to the latter, full-length sequences are biased towards those > 1 kb in length. This indicates that short, full-length cDNA sequences may be underrepresented in our assembly and transcriptome data.

### Genome Size Estimate

**Fig 4:**
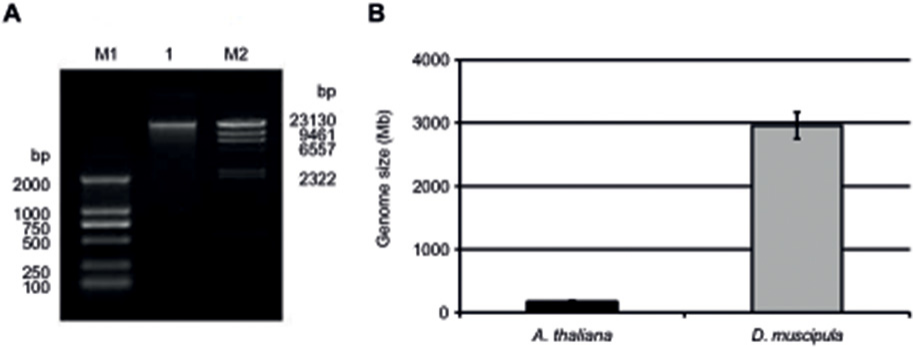
Genomic DNA purification and genome size estimate of *D*. *muscipula*. **(A)** Agarose gel showing a purified fraction of *D*. *muscipula* genomic DNA (1) using a modified CTAB procedure. M1: DNA ladder D2000 (Tiangen), M2: DNA ladder λ-Hind3 digest (Takara). **(B)** Genome size estimate of *D*. *muscipula* using a single-copy qPCR method with *DmACT7* as amplicon. *A*. *thaliana* serves as a control, using *ACTIN1* as amplicon.

An intriguing observation from genome studies of carnivorous plants is the extreme size differences observed among individual family members [11]. To expand the list of genome size estimates of members of the carnivorous orders, we estimated the genome size of *D*. *muscipula*. Using an improved protocol adapted from Bekesiova *et al*. [23], we routinely obtained approx. 25 and 50 μg high quality genomic DNA (gDNA) per g fresh weight from traps and flowers, respectively (Fig 4A).

To estimate the genome size of *D*. *muscipula* using the qPCR-based method of Wilhelm *et al*. [24], a DNA sample without significant RNA contamination is required. From purified gDNA, we targeted the amplification of a single-copy genic region assembled and validated (*DmACT7*, see Fig 1) from our *D*. *muscipula* transcript sequencing. With this sequence as query we used BLASTx to identify the closest homologue. This identified *Arabidopsis ACTIN7* (*ACT7*), with total query coverage of 67% and maximum shared identity of 86%. We therefore designated this target *D*. *muscipula* amplicon *DmACT7*. Using this amplicon, the genome size for *D*. *muscipula* was estimated to be 2956 Mbp (SEM = 210 Mbp, n = 11), equivalent to 3.02 +/- 0.21 pg for the 1C haploid genome (Fig 4B, Table 3). As a control, we estimated the genome size of the model angiosperm *A*. *thaliana* using its *ACTIN1* (*ACT1*) genic region as amplicon. This estimate of 173 Mbp (SEM = 21 Mbp, n = 7; Fig 4B and Table 3) overlaps the well-documented value of the *A*. *thaliana* genome of 157 Mbp (0.16 pg: [25,26]).

**Table 3:** Summary of qPCR-based estimates of haploid genome sizes.

Target	Product length (bp)	Calibration curve y = mx+b(R^2^)	Genome size estimate +/- SEM (Mbp)	n	1C +/- SEM (pg)
***ACT1* (At2g37620)** *A. thaliana*	116	3.263X+45.613 (0.995)	173 +/- 21	7	0.17 +/- 0.02
***ACT7 D. muscipula***	185	3.323X+36.6 (0.994)	2956 +/- 210	11	3.02 +/- 0.21

## Discussion

To date, the highest diversification rates among angiosperms are found in the order Lamiales [27]. In particular, the apparent plasticity observed in the large Lentibulariaceae family has been analyzed [11,13]. In this carnivorous family, three taxa exhibit significantly lower 1C-values than the 157 Mbp of *A*. *thaliana*. These are *Genlisea margaretae* with 63 Mbp, *G*. *aurea* with 64 Mbp, and *Utricularia gibba* with 88 Mbp [11]. Our size estimate for the *Droseraceae* family member *D*. *muscipula* is 46-fold higher than that of the *G*. *margaretae* genome, and comparable to the genome size estimates for carnivorous pitcher plants [15]. Such estimates enable calculation of the minimum number of high-quality reads required for whole-genome sequencing of *D*. *muscipula* and other Gb-sized genomes from carnivorous plants. A good sequencing coverage should provide reliable information on the evolution of carnivory.

The estimated haploid genome size range from 63 Mbp to >3 Gbp indicate that carnivorous plants have undergone dramatic genome evolution. An explanation for such massive proliferation of genome rearrangements, as observed in plastid genomes of Lentibulariaceae members, may be associated with increasingly relaxed functional constraints due to the heterotrophic lifestyle of carnivorous plants [28–30]. Another explanation is that high nucleotide substitution rates are linked to reactive oxygen species (ROS) generated from the increased respiratory rates needed for the oxidative phosphorylation of ADP to ATP upon movement of trapping devices in carnivorous plants [13,31]. ROS cause oxidation of bases and generation of DNA strand interruptions and thereby increases mutation rates [32]. To further understand such events, increased taxon sampling, focusing on both clades and ecological adaptations, is required. Such efforts should elucidate relationships between heterotrophic lifestyles, mutation rates, and genome sizes.

With respect to the *D*. *muscipula* transcriptome, *D*. *muscipula* shares the greatest sequence similarity to tomato (59.8%, Table 2). This is not a surprise, as tomato is the only species included from the asterids clade, to which *D*. *muscipula* also belongs. However, the assembled transcriptome of *D*. *muscipula* also shares sequence similarities to the rosids clade member *Vitis vinifera* (53.8%, Table 2). The relatively strong sequence similarity between carnivorous species and grapes was also reported in a transcriptome study of the carnivorous pitcher plants *Sarracenia psittacina and Sarracenia purpurea* [20]. Future sequencing data on more asterids and rosids members, including transcriptome comparisons with other carnivorous species [19–21], will help to delineate the intriguing phylogeny and molecular adaptation of carnivorous plants and their ecology.

We note that our cost-effective approach using a normalized library of mixed tissues from trap and flowers was only collected from adult plants. Our data therefore does not cover the whole *D*. *muscipula* transcriptome. Still, it aligned 50–60% of the entire complement of RefSeq entries for several model and crop species. Future studies may address the identification of tissue and developmentally regulated genes by temporal and spatial sampling of tissues under different conditions. At present, our data may be mined for comparative studies and as an annotative tool for whole-genome sequencing and future *de novo* assembly of the *D*. *muscipula* genome.

## Conclusion

In this study, the transcriptome of *D*. *muscipula* was sequenced, *de novo* assembled and functionally annotated. An ORF analysis identified a large number of full-length cDNA sequences. The *D*. *muscipula* transcriptome provides some insight into the molecular processes occurring in a Gb-sized carnivorous plant genome. Abundant representation of processes related to the expression of genes associated with catalytic, antioxidant and electron carrier activities was observed. Future uniform meta-analyses of short-read archives, including cDNA sequences from carnivorous *Utricularia* [19] and *Sarracenia* [20] species will aid studies of carnivorous plants and their ecology. This underlines the importance of further expansion of sequence repositories, especially for non-model organisms, for improved understanding of molecular physiology and evolution related to Darwin’s ‘abominable mystery’.

## Supporting Information

S1 Table *Dionaea muscipula* Oases transcriptome assembly file (FA)

S2 Table Assembly contig alignment to NCBI nr database including the best hits in BLAST m8 output format) (TXT)

S3 Table Oligonucleotide primers and sequences used for assembly validation. (PDF)

S4 Table DmACT7 sequence, including primer locations. (PDF)

S5 Table Complete GO annotation term summary. (PDF)
