# Supplementary material for: Transcriptome and Genome Size Analysis of the Venus Flytrap
Source: PLoS One. 2015 Apr 17;10(4):e0123887. doi: 10.1371/journal.pone.0123887 (PMC4401711; doi:10.1371/journal.pone.0123887)
Supplement: S3 Table — (PDF) [file pone.0123887.s003.pdf]

Supplementary file S3

>DmATP synthase Locus_396_Transcript_1/2_Confidence_1.000_Length_426
ATP synthase GI:109066521
atacgagatgtagaatcaagaggatcaacag**caggatagataccgagctc**agatatctgccgggacaac
acggttgtggcatccaagtgagcaaaggtggttgcaggagcgggatccgtcaaatcatcagcaggcaca
taaatagcttgcacggatgtgatggaacctttttcgtagttgtgatacgctcttgaaggcctccaagg
tcagtagcgagggtaggctggtaccccacagcagaaggaatacgtccaagcaaagcagacacctctgag
ttagcttgggtaaagcggaaaatattgtcaataaatagcagcacgtcttgtccctctgcatcacggaaa
tgttcagccacagtcaagccagtaagac**caacacgggcacgagca**ccaggggctcattcatttgaccg
tagacaagagcg

F: caggatagataccgagctc
R: tgctcgtgcccgtgttg

Amplicon: 359 bp

>DmATPase - Locus_14083_Transcript_1/2_Confidence_1.000_Length_1605
ATPase gi|108708096
AAGCAGTGGTATCAACGCAGAGTACGGGGGGCTTGGAAAGTGACGGACTTGAGTCATTGTGCATCAAGG
AGCAGACACTGACAAATGAAAATGCTGAGAAGGTTGTTGGATGGGCTCTAAGCTATCATTTGATGCAGA
ACACAAATGCGGAGTTAGAGGAGAAATTAGTATTGTCTAGTGAGAGCCTTCAGTATGGGATAGAAATCT
TACAGGCTATTCAAAATGAGTCCAAAAGCTTAAAGAAGACACTGAAGGATGTTGTAACCGAAAATGAGT
TTGAGAAAAGGCTTCTGGCAGATGTTATTCCACCCAGTGACATTGGGGTTACATTTGATGATATTGGTG
CCCTTGAGAATGTGAAGGATACATTGAAGGAGTTGGTGATGCTGCCATTGCAGAGGCCGGAACTTTTCT
GCAAGGGACAGTTAGCTAAGCCTTGCAAGGGCATACTTCTGTTTGGCCCTCCTGGCACTGGAAAGACTA
TGCTTGCAAAAGCTGTTGCAACAGAAGCCGGTGCGAACTTTATAAATATTTCCATGTCAAGCATCACAT
CTAAGTGGTTTGGTGAGGGTGAGAAATATGTGAAGGCTGTCTTCTCTCTGGCAAGCAAGATTTCCCCTA
GTGTTGTGTTTGTGGATGAGGTTGATAGTATGCTCGGTCGAAGGGAGAACCCTGGAGAGCATGAGGCCA
TGCGTAAAATGAAAAATGAATTTATGGTGAATTGGGATGGGCTGCGGACTAAAGATACAGAAAGAGTCC
TTGTACTTGCAGCCACTAATAGGCCTTTTGACCTTGATGAAGCGGTCATTAGAAGATTGCCGCGTAGGT
TAATGGTTAACTTGCCAGATGCTCCAAATAGAACTAAGATTCTTAAGGTGATATTGGCAAAAGAAGAAT
TATCTCATGATGTAGATTTAGATGCAGTTGGAAGCATGACCGAGGGATGTTCTGGGAGTGACCTCAAGA
ATCTTTGTGTTGCTGCTGCCCACCGCCCTATCAGGGAGATTCTAGAAAAGGAAAAAAAGGAGGCTGAAG
CTGCTGTGGCTGAAGGTAGACCCGCGCCACCTCTCAGTGGGAGTGCCGATATCCGGCCTCTTAACATGG
ATGACTTCAAACACGCACATGAGCAGGTTTGTGCAAGTGTATCATCGGAGT**CCCATAACATGACCGAGC**
TTCAACAATGGAATGAACTATACGGCGAGGGCGGCTCTAGGAAGAAGGCGGCTCTTAGTTACTTTATGT
AAATGCTTAATATTTCTAGTTATGTGAAAATGTATGTTCTCGGTGGTCGTTTTGTTTTGGCCCGTGATG
TGTATGCCGACGGCTCTGTCTTAGTGGAATTTTTTGTCATGTTTTTTAGGAGGTTTGATACAAGGTAAG
CGTGAGGCCCTTTTGTTTAGGTTTGGCTCACCCTGTATGAAGGTACTGTTTGTTTGTCTGGGGGTATAA
GTTAGTTGAGAGGTATAAAGTTATAAAATAGCGAGGACGGGA**AAGGAGGCGGTGTAGGAACT**GTCATAT
TGCCATTCTTTTATTTTCAGATGTTAATAATATCTTCAGTTCGTCGTTTAAAAAAAAAAAAAAAAGTAC
TCTGCGTTGATACCACTG

F: CCCATAACATGACCGAGCTT
R: AGTTCCTACACCGCCTCCTT

Amplicon 356 bp

>Dm prot. bind. prot.
Locus_17081_Transcript_1/2_Confidence_1.000_Length_2089 protein
binding protein, putative GI:255558542
ATCTAAGCAGTGGTATCAACGCAGAGTACGGGGAGCTGTGTGGACAAGGGTGATAGAAAGGTTGAGGGT
AACAGATTTACTGGGTCACATCATCGTTGGCACGATAATGAAGTAGGACCCTCAGAAGCAATTGATTGT
ACGAAGCTCGCAGTGCAGCTGCACAAGAGTTTTGCTGCTAGAATGGAGGAGGGCTCTTCATTTCAGGTC

GGTAGTTGTGTTGCTACAGTTTCCGGAACTCGTAAAAGGAAAAGCAGATGGGACCAGCCGTCCGATTCA
GAATTTCCTTGCCAGGGACAAAAGGCGTTGCCAATTTTGTGCCAAAATTTTGATTCAAGTCTACACCCT
GAGACGGTCAAGGTTAGACTTCGTCATATAAACAGAGCAAGGCATGTGGAGAAGAGTTCTACTGATTGT
TCTGACGAGCAAACTGAATATTGTACGACAGATAATGGAGTGATGGGCCCTCAGGGAGATGCTCCTCCT
GGATTTTCTTCCCATTTTTCCCCTGGATTCTCTTCTCTTCCTCCTCCTCCTCCTGGGTTTTCTTCCCCT
CTTTGTGGTTCCCATCTTCAGTCATTTGTTGGCTCAACTGTGACTCACATCCCCCAACAGAACAGAAAG
CAGTTGCAATGTTGTAGCCCTTTCCGTGTCTCTTCAGGGCAGCTGCAACG**GAGGTTCAATTCTCGCTTG**
CCTGTAGCATATGGAATTCCATTTTCAGCAATCCAGCAATTTGGGGCACCCCAACGGGGACACCTTGAT
GGTTGGGTTATTGCTCCAGGTATACCTTTTCACCCTTTCCCCCCCTTGCCCACTTACCCCCGTGGGTGC
AACAAGGGAGGTCATCAAGATTCTGGTGCAATGACTGCAGAAAGGGTGAAGGGAAGTGGTAGACAAATG
CCACAAGACCACTGCACACATGTTTCTTATCGAGCAGATCAAGGCATGCCATGCGCCTCAGGTTCGACT
TGTATAGATGTGGTTGCTGCCAG**TACATCCTTTCATCGGCCTC**TGCAGCAGGGAGGAGGCACCTCTTCC
AGCCTGGGAAGGAGGTACTTTAGGCAGCAGAAGTGGAGGAACTCAAAACAAAGGCCGCCGTGGCTGAGA
AGGAACGACTGCGGATTCAAAGGAAACTATCCCCAGAATGGGGTCTACGAGGGTGCAATTCCCAGCGAG
CAGAATGGTCAGTCTATGGAGTATATAAATTATCATGTAGACTGTGTTGGAAATTCTCGATAGCATCGA
ACCGTATCAAGAATACGACATTGATTTAACAATTCTCCAGGGTTCAGAAAAAAAGATTGAAATTTACAT
TTAGATTATAGGAGAGTTGAGGATCTTCTGGATCTTGTCACAGCCACCATAATTCTTTTGTTACATTGA
CGTTGGGGTACACCTTCATCTGAATTTCCTGTTTTTGTTGTAGTTTCGTTATACATTAGAGACATAATT
ATATGTGAATTTTAAAGAGAGATGAAGTTGACCAACCTTGTGGTGCTTCACCAACAAGAAAGGTGAGGT
TATATATTTGAACCTCTCACAAGTAGCAAACAATAAAATTCTCCAAATTCTATTTACCAGCTAGTTTAT
TAAAACCAAAACCTTCTAGTAAGGAACATATGAAGTTTTGCATGTAGAGCAGCACTCATCCGGTTGAGT
ACCGGCGACAAGAATCTGAAGCCAATTTATTGCCCAGAAAAGCAGTCTTCTGCGGTTCATGTATCTGGC
ATTTGCAGGATGTGAGAGAGCATGTTCAGTTTGGTTGCAGAACCATCAACATGTGGATCAGAAATTCTA
TCAATGCTTAGCCTTTGAGGACATTTGTGTTAACTTGTTCCCACGATTTGTGACAGCAGGATTGAGCTC
GGGTAAATTTGAGTACATTTGACTTGAGCCAGGCCTTTTTAACTGATATGATTTATTGTTTACTGTTTT
TTTTGGCCATTTCAATCTAACATGACACCCTTTTAGCCGTACTATCCAGTTGTTTTTTCGTGTAAGCTT
CCAAAATTCCCACAACAAGTGAAGGATCCTTCTTGATAAGCTCACCATAATCATCTCTGTTCATAAACT
TGTCCATGTTATCGGTGAT

F: GAGGTTCAATTCTCGCTTGC
R: GAGGCCGATGAAAGGATGTA

Amplicon : 338 bp

>DmWRKY3 Locus_5911_Transcript_3/3_Confidence_0.600_Length_1263 WRKY DNA-binding protein 3 GI:15227612
TCCACGTCCTATGGTGCCGCCACCACCACAGTGTGAAATGGCTCAAATGGCCGCTCCTTCAAACTTAGT
CCCTAAGGTGGTGGAAGAAGATCCTAAAACTTCAGCAACTTCGGGTAATGCAGATAGACCCTCCTACGA
TGGGTATAACTGGAGAAAATATGGTCAAAAGCAGGTCAAAGGAAGCGAATACCCGAGAAGCTACTACAA
GTGTACGCATCCAAACTGTCCAGTTAAAAAGAAGGTAGAAAGGTCGTTAGATGGGCAAATAGCAGAAAT
TGTCTACAAGGGAGAACACAACCATCCAAAGCCACAGCCCCCCAAGCGCAGTTCTTCGGGAGTGCAAGG
ACAAGGTTCAGTGGCTGATGAGGTAGTCCAGGATCAAGATGGAACTGCCACTGGCACTGGCACTGCCAC
TAATACCAAGTGGAATATTGGTATTGTCAATGCAACTACTGAAGCTTTTGAAGGGCGATTAGAGAACCA
AAATGAAGTAGGATTGTCGACACAGTCAACTCATTCAAACAAGGCCGATTTTGTGCCTTTTGATCCTCT
TGCTGCTAGCAATGGAGATGCTGATACTTGTGGTGTAAGCACTGATTTTGAAGAAGGTAGCAGGGGATT
GGACGTCGATAATGATGAACCAAAAAGCAAGAAGAGGAGAAAAGATGGTCAAAACAATGAAGCAGGACC
GAGCGGAGATGGTGTGCAAGTGCAAGATCCTCCTCGTCATCTTCAAGTGCAAAGCACCACGGAACCTGA
GAGTTTAGGGGACGG**CTTTCGCTGGAGAAAATATGGCCAG**AAGGTCGTTAAAGGAAACCCGTATCCTAG
AAGTTACTACAGATGCACGAGCCTCAAATGCAACGTGCGAAAGCATGTAGAAAGAGCATCCGATGATCC
AAGATCATTCATCACCACGTACGAGGGGAAACACAACCACGAGATGCCCATGAAAAGTACAAATTCAGC
GGCGGCCTCCGAGCCAGATTCATCACAGCTCCTTTCTACAAAGGACAAGAAGTGATTGACCTCACGGTG
ACTACTACATGTTTAACCACTAAACAGTAAACACACCCCTGATGCCAGTCTTTTAATCATTATATAGTT
TTTGTGGTGATATAGAAGCCTAATCAGCAAGTTCTCACTAGTCTGATTTATATATCATCACCACTGAAA
TTCACTGTTTATCATCCTGTTCG**CTACTAGAATCGTAAATTTGCTTA**GCTTATGCAAAAAAAAAAAAAA
GTACTCTGCGTTGATACCACT

F: CTTTCGCTGGAGAAAATATGGCCAG
R: CTACTAGAATCGTAAATTTGCTT

Amplicon: 446 bp

>DmACT7_I 1394 bp
TCGTCATCTCACTCTGCAGGTATATAGAGAATGGCCGATGCTGAGGAGATTCAACCTCTTGTCTGTGAC
AATGGAACTGGTATGGTGAAGGCTGGGTTTGCTGGCGATGATGCTCCTAGGGCAGTGTTTCCCAGTATT
GTTGGGCGTCCCAGGCACACAGGTGTGATGGTTGGTATGGGACAGAAGGATGCTTATGTGGGTGATGAA
GCTCAATCTAAAAGAGGTATCCTTACCTTGAAATACCCCATTGAGCATGGCATTGTCAGCAACTGGGAT
GACATGGAGAAGATCTGGCATCACACTTTCTACAACGAGCTCCGTGTTGCTCCTGAGGAGCATCCGGTG
CTTCTAACTGAGGCTCCTCTCAACCCTAAGGCAAACAGGGAAAAGATGACTCAAATCATGTTTGAGACA
TTCAATGTCCCTGCCATGTATGTTGCTATCCAGGCTGTTCTTTCTCTCTATGCCAGTGGTCGTACAACG
GGTATCGTGTTGGACTCTGGTGATGGTGTGAGTCACACTGTCCCCATTTATGAAGGTTATGCACTTCCC
CATGCTATCCTTCGGCTGGACCTTGCTGGCCGCGACCTCACTGATTCTCTTATGAAGATTCTTACCGAG
AGGGGCTACATGTTCACAACCA**CTGCTGAACGGGAAATTGTT**CGCGACATCAAGGAGAAGCTTGCATAT
GTAGCTCTTGACTATGAGCAGGAGCTGGAAACTGCCAAGAGCAGCAAGTTATTACCATAGGGGCTGAGA
GGTTCAGATGCCCTGAAGTTCTCTTCCAGCCTTCTTTGATTGGGATGGAAGCTGCTGGCATTCATGAGA
CAACCTACAATTCTATCATGAAGTGCGACGTTGATATCAGGAAGGACTTGTATGGTAACATCGTGCTTA
GTGGTGGTTCTACTATGTTCCCTGGCATTGCAGACAGGATGAGCAAGGAAATCACAGCACTTGCTCCAA
GCAGCATGAAGATCAAGGTGGTTGCTCCTCCAGAGAGGAAATACAGTGTCTGGATTGGAGGATCAATCC
TTGCATCTCTCAGCACCTTCCAACAGATGTGGATTTCCAAGGGCGAGTACGATGAGTCTGGTCCATCCA
TTGTCCACAGGAAATGCTTCTAAGCTCTACAGGATGCTTCGAGGGTGAGAGTCCAATATTTTCTTTAGT
TGCCTTGTTGTGTCAAGTGTCATGAACTCGATTCGGTTGAGCTGGAGGATCACGTTGGGTGTGGGTCAT
TGGAAGAAG**GGtgtgcccttgatatgctt**gttatatcaaatatccttcttccagctttcatggaaagtg
cttgatggtactgcatatttttaccttctgtgagctggtcctcacgtagcttttcgccatggctcgact
agtgcttgcgtaga

F: ctgctgaacgggaaattgtt (211)
R: aagcatatcaagggcacacc (203)

Amplicon: 628 bp

>DmCDC48 - Locus_2163_Transcript_2/4_Confidence_0.375_Length_351
Cell division cycle protein 48, putative, GI:110289141
agcaggatcaatgatatctggtctgttagtggcaccaataataaatacagttttcttggcagacatgcc
atccatttcagtgagaagctggtttaaaacacggtccgccgcaccaccagcatcacccacactgcttcc
cctctgtgtagcaattgaatcgagttcatcaaagaataggacacaaggagctgatgcacgagccttatc
gaagatctcacgcacatttgcttcactctccccaaaccacattgtaagcaattcaggtccctttatact
aatgaagtttgcctgacattcatttgcaatagccttggccaacaaagtttttccacaaccaggtgggcc
ataaaa

F: agcaggatcaatgatatctggtc
R: ctttgttggccaaggctattgc

Amplicon: 323 bp

>DmATG7 - Locus_1255_Transcript_1/7_Confidence_0_682_Length_969
CGATCTAAGCAGTGGTATCAACGCAGAGTACAGGGGGAACCTAAGGTATCTCAACACCAGTAGGCAATA
ATTCAACATAGTACAGCCCCTCTCCTTCTCCTCCCCCGGTCCGCGGTGAAATTCGCACTCTACAGTCTA
CAATTCTTCTCCCTCTTCTTCTTGATTTGATCTCCTTGTTAGACACCATGGCCAAGAGTTCGTTCAAGC
TCCAACACCCCCTTGAAAACGGAGGCAGGCTGAAGCTGCACGGATCAGGGAGAAATATCCTGACAGGAT
CCCTGTTATTGTGGAAAAGGCTGAAAGAAGTGACATTCCGGACATTGACAAGAAGAATGACGTTGATGA
ATGTAGATATCTGGTTCCTGCTGACTTAACC**GTGGGGCAGTTTGTCTATGTG**GTAAGGAAGAGGATAAA
GCTCAGTGCCGAGAAAGCTATATTCATCTTTGTGAAGAATTATTCTCCCGCCAACCGCTGCGATGATGT
CTGCTATTTACGAAGAACATAAGGATGAAGATGGATTCCTCTATATGAACTA**CAGTGGTGAGAACACAT**
TTGGTTCATCTTAAGCTCGAGCATTTATGCCGAGTGCATTGTGTATGTAGTAGTGTCCAAGGCAACTAC
TAATTCTGGTGTATATTCTTATCATCTCCCAACTTACCGCAAGTTTCAGTACTTGGATACTCATTTCTC
CACTTTAATGTCTCTTATGGTGATGGTTAATGCAACATGTATATCGTTGTATATCTCTGATTACTTCAT
CTTGGTTAATCCTTTCCTGACAAATTACACGAGCATTTATAAAGGAAACGATGACCAGGGAAAACCTTC
ATGCAGATTGCCAAGTACTGAAAACAGAAACGAAAACAGTGAATCTGCAATAAGTTGAACCACTGAAAA

CATAATGAGAGCCCAGACTACGTCTTTCATGTAAAGGTCCATCAGGAATATTCAATCATAGAGTACAGA
GTA

F: GTGGGGCAGTTTGTCTATGTG (249)
R: CCAAATGTGTTCTCACCACTG (250)

Amplicon: 180 bp

>DmACT7_II 1394 bp
TCGTCATCTCACTCTGCAGGTATATAGAGAATGGCCGATGCTGAGGAGATTCAACCTCTTGTCTGTGAC
AATGGAACTGGTATGGTGAAGGCTGGGTTTGCTGGCGATGATGCTCCTAGGGCAGTGTTTCCCAGTATT
GTTGGGCGTCCCAGGCACACAGGTGTGATGGTTGGTATGGGACAGAAGGATGCTTATGTGGGTGATGAA
GCTCAATCTAAAAGAGGTATCCTTACCTTGAAATACCCCATTGAGCATGG**CATTGTCAGCAACTGGGAT**
GACATGGAGAAGATCTGGCATCACACTTTCTACAACGAGCTCCGTGTTGCTCCTGAGGAGCATCCGGTG
CTTCTAACTGAGGCTCCTCTCAACCCTAAGGCAAACAGGGAAAAGATGACTCAAATCATGTTTGAGACA
TTCAATGTCCCTGCCATGTATGTTGCTATCCAGGCTGTTCTTTCTCTCTATGCCAGTGGTCGTACAACG
GGTATCGTGTTGGACTCTGGTGATGGTGTGAGTCACACTGTCCCCATTTATGAAGGTTATGCACTTCCC
CATGCTATCCTTCGGCTGGACCTTGCTGGCCGCGACCTCACTGATTCTCTTATGAAGATTCTTACCGAG
AGGGGCTACATGTTCACAACCACTGCTGAACGGGAAATTGTTCGCGACATCAAGGAGAAGCTTGCATAT
GTAGCTCTTGACTATGAGCAGGAGCTGGAAACTGCCAAGAGCAGCAAGTTATTACCATAGGGGCTGAGA
GGTTCAGATGCCCTGAAGTTCTCTTCCAGCCTTCTTTGATTGGGATGGAAGCTGCTGGCATTCATGAGA
CAACCTACAATTCTATCATGAAGTGCGACGTTGATATCAGGAAGGACTTGTATGGTAACATCGTGCTTA
GTGGTGGTTCTACTATGTTCCCTGGCATTGCAGACAGGATGAGCAAGGAAATCACAGCACTTGCTCCAA
GCAGCATGAAGATCAAGGTGGTTGCTCCTCCAGAGAGGAAATACAGTGTCTGGATTGGAGGATCAATCC
TTGCATCTCTCAGCACCTTCCAACAGATGTGGATTTCCAAGGGCGAGTACGATGAGTCTGGTCCATCCA
TTGTCCACAGGAAATGCTTCTAAGCTCTACAGGATGCTTCGAGGGTGAGAGTCCAATATTTTCTTTAGT
TGCCTTGTTGTGTCAAGTGTCATGAACTCGATTCGGTTGAGCTGGAGGATCACGTTGGGTGTGGGTCAT
TGGAAGAAG**GGtgtgcccttgatatgctt**gttatatcaaatatccttcttccagctttcatggaaagtg
cttgatggtactgcatatttttaccttctgtgagctggtcctcacgtagcttttcgccatggctcgact
agtgcttgcgtaga

F: CATTGTCAGCAACTGGGATG (217)
R: aagcatatcaagggcacacc (203)

Amplicon: 1014 bp

>DmUCH-like - Locus_34_Transcript_9/10_Confidence_0.462_Length_1720
UCH like GI:115447665
TCTAAGCAGTGGTATCAACGCAGAGTACGGGGGAGCCGATAATCAGTCCACTATATTCTTCAGATTTTT
AGTGGATTTGTTCTGTCATCCTGTGAATATGTGCGGCCAATCCCCCACACCCACCTAGTGGGAAGAGAT
CACGTTGTTATCTTTGTGTGATCTTTAATTTATTAGCTTATTTGGATGAGCAAATGCAAAATTGATTGT
TCGATTGAACTCTAGTTCAACACCATTTTCTGTTTGCCATAAGGAAGGAAGCATATGCAATATACATGC
ACACTGATAGATGCATACATTTCAGTTCAAACAAAAGATTGCCACATAACTTCCAGGACAGGACCTGAC
AAAGAAACTCTGGCCAGTCACATGGAAACACGAAAACTCAAACCACTCGGCTTAGTTTTGGGCTAGTCT
CAAAGATCCCTCCCTCCCTATTCGCATGCAGCCTCATTGCCATAAAACTAACTGTAGCTAACTGGCTCA
ACTTATCCTCATGCAAGGCTCAGATTTGTAAACTCAACTCAACTCTGTCGGGCAGGCGGGCGGGCGAAA
CTAAAACCAAATAAGCAGCTTTTGAACGTCAATCTCAATATGTACCTCCGGAGTTCTTTGAGATTGCCA
TCACATTGAAGTTCACGGAGTCGGGGGTTTTCTGGATGATTTGCTTTATGA**CTTTGGTTGCATCCTGCA**
ATAAGGTACTTGGGGAAGATATACCATGACATACTGGTCCTGATCTCCTTCCATCAAGCTCATAAAGAG
CACCATCTACACAGGTGAAGCAAATAAAATGTGTGTCCACGTTATCTGAAGCCTCAGTCTCGCCAGCAC
TAGCAGCTACAGAATGAGCAACTTCCATTTCTCTGTCATTCTCCAGAAATGCAGCACGCTCCAGAGCAT
CCATGTTTGCTGTGGACTTGAAAAACCTCTCCAAGAATGAGCCCTCAGAAAAGCTTTATTGAAGTGAAG
TTTCCTACAGCATGTAGCAGTCCAATGGTTCCACAAGCATTTCCTACAGTTTGCTTCATGAAATAAACT
TTACTGCTGACATCCTTCTTGATGCTATCCTCCTTCACTCTTTCTGCTTCACTCTCAGGGGTGATGGGA
AAAAGAAAAACCACTGCTAAGACAGGCTTTGGAACCATTTCCAGTAATTCATCATCCAAGCCATAAACA
TCATAGCACTCTGCTTCCTCTACTGGAAGACCAAGCCCCCAGAGAAACTGGTTCATGACATCCGGGTT**A**
GCTTCGAGAGGAAGCCACCTTTTCGATGAAGGGTTTTCGTCCATGGCGGAATTGGGACTCTCTCTCTCT
CTCTGTCGCCTACGTGCCTCTGCTTGCTTGTCCTTCTCTTCTTTCTTCTTTGAAGAAAGAGTTGCAAAG

ACCTTCTCATGATGGATGGCCCTCGGAGGTTTTGCAGTCGAAATTGTGACGACAATGTCGATAGTGTTG
GTTCCAGTTCTCGCGTAGCACGCCACATATTATTGTCTTTGCCTGAAGTTCAATTGTATCACAAAAGCA
AACTCGTCCATTATAGAGAACTATTGAGTATGGCTGATACCATGCCCTGAACAAGGTCCGAGTCCTGTG
ATCGGACTTGGGAGACGACGAGGAAAGGCAGAGATCCAATTTGTAGACCTTCCCCATAGCTAGAAGTAT
GTATATGTTGAAGAAATTGCGACACCCCCTTCCATGCCTTCTCCTCCTCCTGCTACTGCAGTCT

F: CTTTGGTTGCATCCTGCAATAAGG
R: AGCTTCGAGAGGAAGCCAC

Amplicon: approx. 589 bp

>DmUBQ - Locus_4158_Transcript_3/4_Confidence_0.700_Length_1310 ubq
GI:102655942
AAAGGGGGAGAGAAGTACTACAGCTGCAGAACATTTATGCAAAACGCATTTGTATGGAGAAACCAGAAT
TGAACAAATTAAAGTACAGGTAGAACACACACTTATTAAATCCACAATATTTGGACCCACAATTTCGAA
AGAGAGTCACTAAAACATGGCTAAATCACACAATATACTGATATATATCAAGAGGTAAGATGAAAGACA
TATAATAGCAAATGGTGGCTGGGCTGCAATCAAATTCTCAGAAATCACCTCCACGAAGGCGGAGGACGA
GATGAAGAGTTGATTCCTTTTGGATGTTATAGTCGGCTAGGGTTCGGCCATCTTCCAACTGCTTCCCGG
CAAAGATGAGCCTCTGCTGGTCCGGAGGAATTCCTTCCTTGTCCTGAATTTTGGACTTCACATTATCAA
CGGTGTCCGAGCTCTCCACCTCCAAAGTGATGGTCTTCCCAGTAAGAGTCTTAACAAAGATCTGCATCC
CACCACGGAGCCTCAGGACAAGGTGAAGCGTAGACTCCTTCTGAAGTTGTAATCCACGAGCGTACGGCC
ATCCTCGAGCTGTTTGCCAGCAAAGATCAGCCTCTGTTGGTCTGGAGGAATGCCCTCTTTGTCCTGAAT
CTTTGCCTTCACATTGTCAATTGTGTCAGAGCTCTCGACCTCCAATGTGATGGTCTTGCCGGTGAGA**GT**
CTTCACAAAGATCTGCATACCACCACGGAGACGAAGAACAAGGTGGAGTGTTGATTCTTTCTGGATGTT
ATAGTCGGCAAGCGTACACCATCTTCAAGCTGTTTCCCAGCAAAGATGAGCCTCTGCTGATCTGGAGGA
ATGCCCTCCTTATCCTGAATCTTGGCCTTCACATTGTCAACTGTGTCAGAGCTCTCCACTTCCAGGGTG
ATTGTCTTG**CCAGTGAGGGTCTTGACAAAGAT**TTGCATCCCCCCACGGAGGCGGAGGACAAGGTGAAGA
GTTGATTCTTTCTGGATGTTGTAGTCTGCTAGGGTTCGGCCATCTTCAAGCTGTTTCCCAGCAAAGATC
AGCCTTTGCTGATCTGGGGGAATTCCCTCCTTATCTTGGATCTTTGCTTTCACATTGTCAATGGTGTCA
GAGCTCTCAACCTCAAGAGTGATCGTCTTCCCCGTTAGAGTCTTAACAAATATCTGCATCTTTTAGCAA
GAAAAGGAGAGAGAACAAGAACGGCGAGCAAACGGGGAAGCTGATCCGATCAACGACTCTGTGAAGTGT
GAATACGACCTCCGGGGATGGTGAATTGATTGCCCCCCCGTACTCTGCGTTGATACCACTGCTTAG

F: GTCTTCACAAAGATCTGCATACCACC
R: ATCTTTGTCAAGACCCTCACTGG

Amplicon: 247
