# Supplementary material for: Transcriptome and Genome Size Analysis of the Venus Flytrap
Source: PLoS One. 2015 Apr 17;10(4):e0123887. doi: 10.1371/journal.pone.0123887 (PMC4401711; doi:10.1371/journal.pone.0123887)
Supplement: S4 Table — (PDF) [file pone.0123887.s004.pdf]

Supplementary file S4

\>DmACT7_II 1394 bp
TCGTCATCTCACTCTGCAGGTATATAGAGA**ATG**GCCGATGCTGAGGAGATTCAACCTCTTGTCTGTGAC
AATGGAACTGGTATGGTGAAGGCTGGGTTTGCTGGCGATGATGCTCCTAGGGCAGTGTTTCCCAGTATT
GTTGGGCGTCCCAGGCACACAGGTGTGATGGTTGGTATGGGACAGAAGGATGCTTATGTGGGTGATGAA
GCTCAATCTAAAAGAGGTATCCTTACCTTGAAATACCCCATTGAGCATGG**CATTGTCAGCAACTGGGAT**
GACATGGAGAAGATCTGGCATCACACTTTCTACAACGAGCTCCGTGTTGCTCCTGAGGAGCATCCGGTG
CTTCTAACTGAGGCTCCTCTCAACCCTAAGGCAAACAGGGAAAAGATGACTCAAATCATGTTTGAGACA
TTCAATGTCCCTGCCATGTATGTTGCTATCCAGGCTGTTCTTTCTCTCTATGCCAGTGGTCGTACAACG
GGTATCGTGTTGGACTCTGGTGATGGTGTGAGTCACACTGTCCCCATTTATGAAGGTTATGCACTTCCC
CATGCTATCCTTCGGCTGGACCTTGCTGGCCGCGACCTCACTGATTCTCTTATGAAGATTCTTACCGAG
AGGGGCTACATGTTCACAACCACTGCTGAACGGGAAATTGTTCGCGACATCAAGGAGAAGCTTGCATAT
GTAGCTCTTGACTATGAGCAGGAGCTGGAAACTGCCAAGAGCAGCAAGTTATTACCATAGGGGCTGAGA
GGTTCAGATGCCCTGAAGTTCTCTTCCAGCCT**TCTTTGATTGGGATGGAAGC**TGCTGGCATTCATGAGA
CAACCTACAATTCTATCATGAAGTGCGACGTTGATATCAGGAAGGACTTGTATGGTAACATCGTGCTTA
GTGGTGGTTCT**ACTATGTTCCCTGGCATTGC**AGACAGGATGAGCAAGGAAATCACAGCACTTGCTCCAA
GCAGCATGAAGATCAAGGTGGTTGCTCCTCCAGAGAGGAAATACAGTGTCTGGATTGGAGGATCAATCC
TTGCATCTCTCAGCACCTTCCAACAGATGTGGATTTCCAAGGGCGAGTACGATGAGTCTGGTCCATCCA
TTGTCCACAGGAAATGCTTCTAAGCTCTACAGGATGCTTCGAGGG**TGA**GAGTCCAATATTTTCTTTAGT
TGCCTTGTTGTGTCAAGTGTCATGAACTCGATTCGGTTGAGCTGGAGGATCACGTTGGGTGTGGGTCAT
TGGAAGAAG**GGtgtgcccttgatatgctt**gttatatcaaatatccttcttccagctttcatggaaagtg
cttgatggtactgcatatttttaccttctgtgagctggtcctcacgtagcttttcgccatggctcgact
agtgcttgcgtaga

Inner forward primer: tctttgattgggatggaagc

Inner reverse primer: gcaatgccagggaacatagt

Outer forward primer: cattgtcagcaactgggatg

Outer reverse primer: aagcatatcaagggcacacc
