# Supplementary material for: Transcriptome and Genome Size Analysis of the Venus Flytrap
Source: PLoS One. 2015 Apr 17;10(4):e0123887. doi: 10.1371/journal.pone.0123887 (PMC4401711; doi:10.1371/journal.pone.0123887)
Supplement: S5 Table — (PDF) [file pone.0123887.s005.pdf]

Supplementary file S5. Complete GO annotation term summary

Cellular Component:
17 (0.2) GO:0005576 extracellular region
3 (0.0) GO:0044421 extracellular region part
12 (0.1) GO:0048046 apoplast
2588 (26.3) GO:0005623 cell
2588 (26.3) GO:0044464 cell part
3 (0.0) GO:0019012 virion
3 (0.0) GO:0044423 virion part
72 (0.7) GO:0031974 membrane-enclosed lumen
6 (0.1) GO:0031970 organelle envelope lumen
66 (0.7) GO:0043233 organelle lumen
58 (0.6) GO:0031975 envelope
8 (0.1) GO:0030313 cell envelope
51 (0.5) GO:0031967 organelle envelope
746 (7.6) GO:0032991 macromolecular complex
312 (3.2) GO:0030529 ribonucleoprotein complex
9 (0.1) GO:0032993 protein-DNA complex
426 (4.3) GO:0043234 protein complex
1017 (10.3) GO:0043226 organelle
25 (0.3) GO:0031982 vesicle
659 (6.7) GO:0043227 membrane-bounded organelle
370 (3.8) GO:0043228 non-membrane-bounded organelle
1017 (10.3) GO:0043229 intracellular organelle
316 (3.2) GO:0044422 organelle part
3 (0.0) GO:0044421 extracellular region part
3 (0.0) GO:0031012 extracellular matrix
316 (3.2) GO:0044422 organelle part
121 (1.2) GO:0031090 organelle membrane
66 (0.7) GO:0043233 organelle lumen
316 (3.2) GO:0044446 intracellular organelle part
3 (0.0) GO:0044423 virion part
3 (0.0) GO:0019028 viral capsid
2588 (26.3) GO:0044464 cell part
1575 (16.0) GO:0005622 intracellular
3 (0.0) GO:0008287 protein serine/threonine phosphatase complex
2 (0.0) GO:0009349 riboflavin synthase complex
96 (1.0) GO:0012505 endomembrane system
1177 (12.0) GO:0016020 membrane
1 (0.0) GO:0019008 molybdopterin synthase complex
55 (0.6) GO:0030312 external encapsulating structure
8 (0.1) GO:0042597 periplasmic space
1374 (14.0) GO:0044424 intracellular part
658 (6.7) GO:0044425 membrane part
8 (0.1) GO:0044462 external encapsulating structure part

Total: 43

Biological Process:
132 (1.3) GO:0044085 cellular component biogenesis
6 (0.1) GO:0032502 developmental process
20 (0.2) GO:0000003 reproduction
201 (2.0) GO:0016043 cellular component organization
10 (0.1) GO:0016265 death
20 (0.2) GO:0022414 reproductive process
285 (2.9) GO:0050896 response to stimulus
14 (0.1) GO:0032501 multicellular organismal process
85 (0.9) GO:0010926 anatomical structure formation
22 (0.2) GO:0051704 multi\-organism process
733 (7.4) GO:0051234 establishment of localization
5 (0.1) GO:0022610 biological adhesion
5136 (52.2) GO:0008152 metabolic process
649 (6.6) GO:0043473 pigmentation
753 (7.7) GO:0051179 localization
4285 (43.6) GO:0009987 cellular process
681 (6.9) GO:0065007 biological regulation

Total: 17

Molecular Function:
184 (1.9) GO:0009055 electron carrier activity
86 (0.9) GO:0060089 molecular transducer activity
212 (2.2) GO:0030528 transcription regulator activity
128 (1.3) GO:0030234 enzyme regulator activity
4847 (49.3) GO:0003824 catalytic activity
5348 (54.4) GO:0005488 binding
56 (0.6) GO:0016209 antioxidant activity
2 (0.0) GO:0016530 metallochaperone activity
14 (0.1) GO:0045735 nutrient reservoir activity
44 (0.4) GO:0045182 translation regulator activity
326 (3.3) GO:0005198 structural molecule activity
423 (4.3) GO:0005215 transporter activity

Total: 12

==
Total GO terms in three ontologies: 72
